# Sharp increase in *Chlamydia pneumoniae* infections in 2024 in Germany

**DOI:** 10.1007/s10096-025-05244-z

**Published:** 2025-08-30

**Authors:** Sébastien Boutin, Andi Krumbholz, Tobias Siegfried Kramer, Michael Boehm, Norbert Schmeisser, Rolf Kaiser, Jan Rupp

**Affiliations:** 1 Institute of Medical Microbiology, University of Lübeck and University Hospital Schleswig-Holstein, Campus Lübeck, Lübeck, Germany; 2 German Center for Infection Research (DZIF), Partner Site Hamburg-Lübeck-Borstel-Riems, Lübeck, Germany; 3 Airway Research Center North (ARCN), German Center for Lung Research (DZL), Lübeck, Germany; 4 Labor Dr. Krause und Kollegen Medizinisches Versorgungszentrum GmbH, Kiel, Germany; 5 Institute of Medical Microbiology, University of Kiel and University Hospital Schleswig-Holstein, Campus Kiel, Kiel, Germany; 6 LADR der Laborverbund Dr. Kramer & Kollegen, Geesthacht, Germany; 7 Institute of Virology, University of Cologne, Cologne, Germany; 8 Medeora, Cologne, Germany; 9 Infectious Disease Clinic, University Hospital Schleswig-Holstein, Campus Lübeck, Lübeck, Germany

**Keywords:** *Chlamydia pneumoniae*, Intracellular bacteria, Respiratory infection, Surveillance

## Abstract

*Chlamydia pneumoniae* (*C. pneumoniae*) is a recognized cause of respiratory infections in children and adolescents, while it is often considered a negligible pathogen in adults outside of outbreaks. We performed a retrospective analysis from a nationwide surveillance network in Germany that collected data from 2018 to 2024. A multivariate analysis (binomial model) was performed to assess the influence of year, hospitalization status, gender and age on *C. pneumoniae* detection rate. Our analysis showed an increasing *C. pneumoniae* detection rate in 2024 compared to 2019, especially in children below 15 years and adults aged 30–50 years, mostly in patients who were treated as outpatients.

## Brief report

The intracellular bacterium *Chlamydia pneumoniae* (*C. pneumoniae*) is a recognized cause of respiratory tract infection. While the infection is asymptomatic or mild in most cases, it can lead to severe respiratory illnesses such as community-acquired pneumonia (CAP), particularly in children [1]. In addition, acute and/or recurrent infections can result in worsening of existing chronic pulmonary diseases such as chronic obstructive pulmonary disease, asthma, and lung cancer [2]. The prevalence of *C. pneumoniae* in CAP was recently estimated to be less than 1.5% [3, 4], so that the pathogen has fallen out of clinical focus in adults. However, recent data from France indicate a significant rise of *C. pneumoniae* infection in 2024 [5]. The report is consistent with own data from the ClinVirolNet surveillance network, showing an increase of *C. pneumoniae* detection rate across 33 laboratories distributed across whole of Germany. We therefore examined the extent of this increase in more detail to understand the factors associated with a *C. pneumoniae-*positive detection.

As *C. pneumoniae* infections are not subject to notification in Germany, the data were collected between 01/2018 and 12/2024 via a voluntary surveillance network of certified medical laboratories called Clinical Virology Network (CVN, https://public.clinical-virology.net/). The network continuously collects data on pathogen detections from diagnostic respiratory samples taken from in- and outpatients presenting with clinical symptoms indicative of an acute respiratory infection (ARI) using various validated and quality-controlled nucleic acid amplification techniques (NAT). Common clinical variables were included in the model: Age, gender, year of testing, and hospitalization status (hospitalized vs. ambulant patients). The study is based on cumulative data collection performed and pseudonymized locally. Demographic data are presented in the Table 1.

**Table 1:** Demographic data of the cohort included in the study

	Year 2018, *N* = 1741		Year 2019, *N* = 3394		Year 2020, *N* = 5429		Year 2021, *N* = 3884		Year 2022, *N* = 8378		Year 2023, *N* = 8955		Year 2024, *N* = 18,864	
*C. pneumoniae* test	**-** *N* = 1,714^#^	**+** *N* = 27^#^	**-** *N* = 3,377^#^	**+** *N* = 17^#^	**-** *N* = 5,425^#^	**+** *N* = 4^#^	**-** *N* = 3,880^#^	**+** *N* = 4^#^	**-** *N* = 8,370^#^	**+** *N* = 8^#^	**-** *N* = 8,939^#^	**+** *N* = 16^#^	**-** *N* = 18,544^#^	**+** *N* = 320^#^
age	46 (14, 68)	18 (15, 25)	57 (22, 72)	21 (14, 56)	62 (40, 75)	39 (39, 39)	61 (40, 73)	NA	45 (8, 68)	40 (9, 58)	33 (5, 65)	22 (9, 54)	37 (8, 65)	11 (8, 32)
gender														
female	843 (49%)	7 (26%)	1,320 (39%)	9 (53%)	1,697 (31%)	0 (0%)	1,197 (31%)	0 (0%)	3,197 (38%)	3 (38%)	3,727 (42%)	7 (44%)	8,205 (44%)	141 (44%)
male	868 (51%)	20 (74%)	2,027 (60%)	7 (41%)	2,821 (52%)	3 (75%)	2,125 (55%)	2 (50%)	4,917 (59%)	5 (63%)	5,207 (58%)	9 (56%)	10,311 (56%)	177 (55%)
unknown	3 (0.2%)	0 (0%)	30 (0.9%)	1 (5.9%)	907 (17%)	1 (25%)	558 (14%)	2 (50%)	256 (3.1%)	0 (0%)	5 (< 0.1%)	0 (0%)	28 (0.2%)	2 (0.6%)
hospitalization status														
ambulant	583 (34%)	10 (37%)	1,241 (37%)	12 (75%)	890 (16%)	1 (25%)	514 (13%)	2 (50%)	771 (9.2%)	0 (0%)	2,047 (23%)	8 (73%)	8,395 (45%)	240 (75%)
hospitalized	1,131 (66%)	17 (63%)	2,135 (63%)	4 (25%)	4,535 (84%)	3 (75%)	3,366 (87%)	2 (50%)	7,599 (91%)	8 (100%)	6,892 (77%)	3 (27%)	10,149 (55%)	80 (25%)

^#^ Median (Q1, Q3); n (%)

We performed a logistic regression model (binomial family, link logit) including all four variables to understand the factors associated with a positive NAT test for *C. pneumoniae*. To minimize the influence of multiple testing, only the first test was used for each patient, unless the patient’s infection status changed due to a positive NAT test. This situation occured in 201 patients for whom the second test was also included to consider that the patient became positive at a later time point. Following this procedure, a total of 72,327 cases were available from 50,444 patients but it was reduced to 50,645 cases (70.0%) for the analysis. All statistics and figures were performed using R software version 4.5.1.

**Fig. 1:**
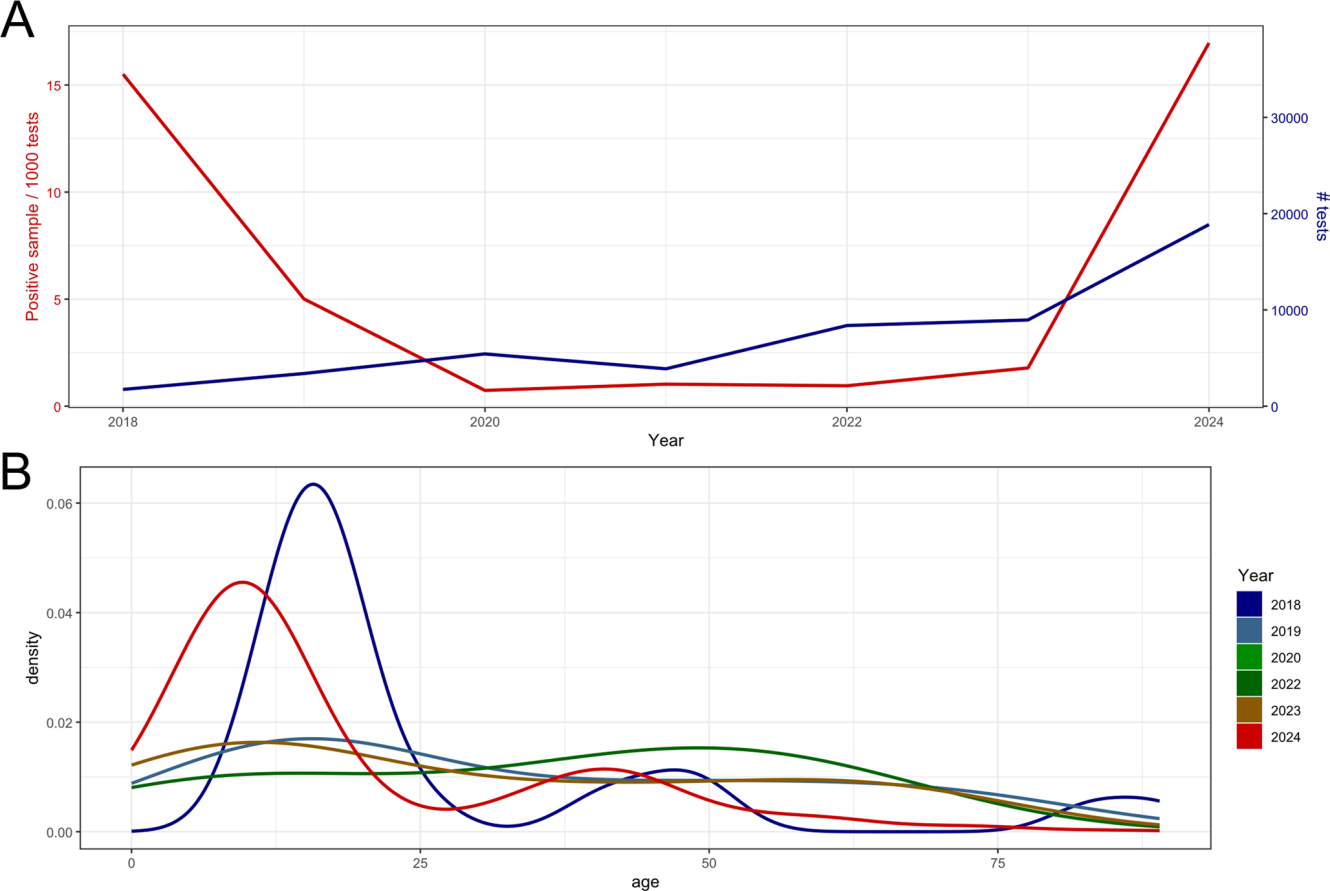
Increase in the detection rate of *C. pneumoniae*. In panel **A**, we depict the total number of tests (in blue, scale on the right y-axis) and positive sample/1000 tests (in red, scale on the left y-axis), and in panel **B**, the density plot of the positive tests per age stratified by the time period

We observed a first small peak in the *C. pneumoniae* detection rate in 2018 with 15.5 positive cases per 1000 tests performed (adj. OR: 3.52, p-value < 0.001) so we used the year 2019 as a reference for the logistic model in 2019. During 2019 until 2023 we observed a lower but stagnating detection rate from 0.72 to 5.01 of *C. pneumoniae*-positive cases per 1000 tests followed by a significant increase in the positivity rate in 2024 with 16.96 positive cases per 1000 tests performed (adjusted OR : 3.03, *p* < 0.001; Table 2; Fig. 1A).

**Table 2:** Logistic regression predicting the positivity rate of *C.* *pneumoniae*

Logistic regression	adj. OR (95%CI)	*P*-value (Wald’s test)
Year: ref.=2019		
2018 2020 2021 2022 2023 2024	3.52 (1.82,6.8) 0.06 (0.01,0.49) 0 (2.19e + 225) 0.22 (0.08,0.57) 0.29 (0.13,0.64) 3.03 (1.76,5.19)	< 0.001 0.009 0.957 0.002 0.002 < 0.001
age (cont. var.)	0.98 (0.97,0.98)	< 0.001
gender: ref = Female		
Male	1.11 (0.9,1.37)	0.329
Unknown	2.43 (0.7,8.42)	0.16
hospitalized or ambulant	0.32 (0.26,0.41)	< 0.001

Following the logistic regression, we did not observe an impact of gender on the NAT positivity rate, but hospitalized patients had a lower positivity rate (adjusted-OR: 0.32, *p* < 0.001). Age also had a significant impact on NAT positivity (adjusted-OR: 0.98, *p* < 0.001), with children (0-15yrs) and adults aged 30–50 yrs being the most commonly affected patient group in the year 2018 and 2024 (Fig. 1B).

Our data show that the increase of *C. pneumoniae* infections in 2024 not only affects children and young adults but also adults aged 30 to 50 years. The detection peak from 2018 should be interpreted with caution due to the lower number of tests carried out at that time but coincides with a cluster of cases in children reported in Finland during the same time period [6]. The recent increase in Germany is more comparable to a local outbreak in Switzerland, where a 2:1 ratio of children to adults was observed [7] and correlates with the increased *C. pneumoniae* infection rates in young adults in France that were recently reported by Edouard et al. [5]. Our data suggest that the incidence of infection is predominantly community-based and less likely to be found in hospitalized patients.

It is difficult to provide a direct cause for the recent emergence of *C. pneumoniae* infections. We can exclude that the higher number of NAT tests performed after the COVID-19 pandemic is responsible for the observed increase, as detection frequencies of other non directly transmissible pathogens like Legionella *sp* [8]. that were included in the respiratory test panels were not increased over time (data not shown).

A general decline in mucosal and/or systemic immunity to circulating respiratory infections has been discussed for several pathogens that have re-emerged following the release of COVID-19 pandemic preventive measures. This hypothesis is supported by the sharp decrease in *C. pneumoniae* prevalence in 2019, the lack of *C. pneumoniae* detection at the peak of hygienic measures and the similar infection dynamics that were observed with *Mycoplasma pneumoniae*, Influenza A virus, respiratory syncytial virus and group A streptococci [9, 10]. However, the assumption is limited by the lack of NAT-based detection rates for *C. pneumoniae* over several decades. An increase in infections due to the emergence of a new *C. pneumoniae* strain as suggested by the data from Edouard et al. [5]. can also not be entirely excluded. Previous studies have shown that genetic diversity among the few available *C. pneumoniae* isolates is low [11], but with the new sequencing technologies available, such variations in the prevalence of rare pathogens can be tracked more accurately even in the absence of isolates.

In summary, data from a voluntary surveillance network in Germany show a sharp increase in *C. pneumoniae* detections in 2024 supporting recent findings from Edouard et al. [5] in France. The timely and clinically availability of surveillance data on non-notifiable pathogens of respiratory tract infections indicating epidemiological changes in the pathogen spectrum could have an immediate therapeutic impact. Similar to what was observed during the recent peak of *M. pneumoniae* infections, more effective intracellularly active antibiotics could then be used as first line therapies for a limited period of time.

Overall, key aspects of seasonality, transmission dynamics between children and adults, as well as the clinical relevance are still not well understood in *C. pneumoniae* infections and need further investigations.

## Data Availability

No datasets were generated or analysed during the current study.
